# Antibiotic consumption in long-term care facilities in Poland and other European countries in 2017

**DOI:** 10.1186/s13756-021-01019-1

**Published:** 2021-10-26

**Authors:** Jadwiga Wojkowska-Mach, Michał Brudło, Mariusz Topolski, Tomasz Bochenek, Estera Jachowicz, Małgorzata Siewierska, Anna Różańska

**Affiliations:** 1 Department of Microbiology, Faculty of Medicine, Jagiellonian University Medical College, Czysta 18, 31-121 Krakow, Poland; 2 Faculty of Medicine, Jagiellonian University Medical College, Krakow, Poland; 3 Department of Systems and Computer Networks, Faculty of Electronics, Wrocław University of Science and Technology, Wrocław, Poland; 4 Department of Nutrition and Drug Research, Institute of Public Health, Faculty of Health Sciences, Jagiellonian University Medical College, Krakow, Poland; 5 Department of Ophthalmology, St. Rose Hospital, Krakow, Poland

**Keywords:** Antibacterials, Antibiotic consumption, Care facilities, Long-term, LTCF

## Abstract

**Introduction:**

The aim of this research study was to compare the situation concerning the use of microbiology testing, the epidemiology of healthcare-associated infection (HAI) and antimicrobial consumption (AMC) in Polish long-term care facilities (LTCFs) with other European countries, using the most recent findings available in the European databases. Furthermore, this study aimed to highlight several basic factors that contribute to the observable differences in AMC between countries participating in the HALT-3 study, especially the relationship with demographic indicators, as well as the health care resources utilization rates.

**Patients and methods:**

The most recent HAIs in Long-Term care facilities Point Prevalence Survey (HALT PPS) was carried out in EU/EEA countries in 2016–2017, and in Poland it was carried out in April–June 2017 in 24 LTCFs. AMC data was collected with use of methodology of the Anatomical Therapeutic Chemical (ATC) classification system of the WHO.

**Results:**

In total total in HALT-3 study on the day of the PPS, 5035 out of the 102,301 eligible residents received at least one antimicrobial agent, with prevalence of 4.9%, and in Poland 3.2%. The most common HAIs in the countries included into the study was urinary tract infection with relative frequency of 32%, in Poland it was skin infection, 30.4%. The respiratory tract infections, excluding pneumonia (PNU) were observed in 29.5% of residents in total, in Poland 17.4%, the prevalence rate of PNU were 1.4% and 5.4%, respectively. The lack of microbiological results of HAIs testing concerned the vast majority of all HAIs, 75.8% in total and 81.5% in Poland. The most frequently used antibacterial for systemic use were beta-lactams and the most frequently prescribed antimicrobial agent was ‘amoxicillin and enzyme inhibitor’. AMC was closely correlated with the age of the general population (65 years of age and more) and the availability of doctors in general population.

**Conclusions:**

A significant problem observed in LTCFs was the empirical use of antibiotics and the scarcity of microbiological testing. In the studied Polish LTCFs, where the age of residents was low, also the AMC was found to be lower.

**Supplementary Information:**

The online version contains supplementary material available at 10.1186/s13756-021-01019-1.

## What’s new

The significant problem observed in long-term care facilities (LTCFs) in Poland and other European countries is the empirical use of antibiotics and the scarcity of microbiological testing. Antibiotic consumption in European LTCFs is closely correlated with the age of the general population (65 years of age and more) and the availability of doctors in general population. Antimicrobial consumption (AMC) in LTCFs was found to be lower in Poland than in the overall group of all studied European countries.

## Introduction

Prevalence of multi-drug resistant microorganisms has been increasing across the European countries and currently about 20% of infections in this region is due to antibiotic-resistant bacteria [1].

Unfortunately, one of the strongest contributors to the increasing drug resistance is the consumption of antibiotics, not only in hospital or ambulatory care, but also in long-term care facilities (LTCF).

The frail elderly residents within LTCFs are extremely exposed to the risk of healthcare-associated infections (HAI), due to their co-morbidities, frequent hospitalizations, dependence on care, due to living in a limited space in close contact with other residents [2].

To quantify the phenomenon of HAIs in LTCFs at the European level, the European Centre for Disease Prevention and Control (ECDC) established the project named as the Healthcare-Associated Infections in Long-Term Care Facilities (HALT) in 2010. In May 2015, ECDC launched the third pan-European project HALT-3, updating tools for the survey. The researchers representing Poland participated in this project during the period of April-June 2017.

## Objectives

The aim of this research study was to compare the situation concerning the use of microbiology testing, the epidemiology of HAIs and AMC in Polish LTCFs with other European countries, using the most recent findings available in the European databases. Furthermore, this study aimed to highlight several basic factors that contribute to the observable differences in AMC between countries participating in the HALT-3 study, especially the relationship with demographic indicators, as well as the health care resources utilization rates.

## Patients and methods

The most recent HALT-3 was carried out in the selected European countries in years 2016–2017. In Poland, it was conducted in between April and June 2017, embracing 24 LTCFs (12 residential homes (RH) and 12 nursing homes (NH)). LTCFs taking part of the study were located in central and south-eastern Poland, which does not represent the characteristics of the whole country. All participating LTCFs implemented the study protocols. The coordinating centre for Poland was the Chair of Microbiology of the Jagiellonian University Medical College in Krakow—The persons being the subjects of HALT-3 study, further called “residents”, were defined as persons having the status of residents of LTCFs for a duration of at least 48 h, as counted on the day of study. The nursing home (NH) was defined precisely as the institution where residents receive physician’s or nurse’s care 24 h a day, thus resulting in health care on a level which was more advanced than that provided at RH. The residents of NH are unable to live independently and require assistance at activities of their daily living. Infections were defined according to the McGeer’s criteria [3] and were detected by trained personnel in LTCF on site.

The antimicrobial consumption data was collect using the methodology of the Anatomical Therapeutic Chemical (ATC) classification system of the World Health Organization Collaborating Centre for Drug Statistics Methodology [4]. All oral, rectal, intramuscular, and intravenous treatments with antibacterials for systemic use, as well as inhaled antibiotic treatments were included in this study, while treatments with antimicrobial agents for topical use were excluded.

The studied outcomes were: (1) the prevalence of residents that have had at least one HAIs on the day of survey and (2) the prevalence of antimicrobial consumption (AMC) expressed as the number of residents with antimicrobials on the day of survey divided by the number of residents in LTCFs multiplied by 1000. The survey was performed in 24 EU/EEA (European Union/European Economic Area) countries and two EU candidate countries. In total, 5035 facilities with 103,763 eligible residents from 26 EU/EEA countries participated in the survey, including 24 facilities and 2281 residents from Poland [5]. The number of residents included into the survey constitutes 1.6% of all residents of LTCFs in Poland [6]. On average (for all participating countries) 45.9% were general NHs; 12.3% RHs; 22.3% mixed-type facilities and 19.5% others. In Poland, 50% of all settings were general NHs and 50% were RHs. In total, 5098 prescriptions of antibacterials for systemic use (ATC J01) were reported. The observed AMC prevalence for the EU was 4.9% (95%CI 4.8 to 5.1), and in Poland 3.2% (95% CI 2.5 to 4.0). For all countries the main indication for antimicrobial use was treatment (69.5%), more often than prevention (29.4%), while unknown use was observed for 1.1% of prescriptions. In Poland it was 90% and 10% for treatment and prevention respectively [7].

The statistical analysis of relationship between selected AMC and demographic indicators, as well as health care resources utilization rates, was performed for the selected countries, using publicly available data. The sources of these data, their characteristics, and application in this study are presented in Additional file 1: Table S1.

### Statistical analysis

Within the statistical analysis of the study results, several basic statistics, such as the number and percentage were calculated. Relationships between the variables were calculated with the use of Spearman's rho correlation coefficients. All tests were calculated at the level of statistical significance alpha = 0.05. Data in the so-called preprocessing was also verified for consistency of results with respect to the included countries. In this way, it was possible to show whether the given results differ from the others, and thus constitute different relations. For this purpose, two methods of selecting and extracting traits were used: CCPCA (Centroid Class Principal Components Analysis) [8] and GPA (Gradient Principal Components Analysis) [9]. It was found that the parameters analyzed constitute a consistent construct in all analyzed EU/EEA countries (Table 3). The consistency of the data demonstrated by both methods allows for drawing conclusions about the correlations between the variables for these countries with an accuracy of 95%, thus justifying the conclusion that the results are reliable.

### Ethical approval

The study HALT-3 for Poland has received a positive opinion of the Bioethical Commission of the Jagiellonian University in Krakow (no. 122.6120.64.2017 of 20.06.2017).

## Results

Overall, on the day of the PPS, 5035 of the 102,301 eligible residents received at least one antimicrobial agent (5088 prescriptions) with prevalence 4.9%, in Poland 73 residents out of the total number 2281 residents, giving the prevalence of 3.2%. Generally, the most common HAIs in the countries included into the study was urinary tract infection (UTI) with relative frequency of 32%, while in Poland the most common HAIs was skin infection (30.4%, Table 1). The respiratory tract infections, excluding pneumonia (PNU) were observed in 29.5% of residents in total, but in Poland 17.4%, the prevalence rate of PNU were 1.4% and 5.4%, respectively.

**Table 1:** The prevalence of healthcare associated infections in residents of long-term care facilities with at least one healthcare associated infections on the day of the point prevalence surveys, by type of infections, 2016–2017

Type of HAI	HALT PPS total n (%)	HALT PPS Poland n (%)
All HAIs	3.858 (100.0)	92 (100.0)
UTI	1.233 (32.0)	27 (29.3)
Respiratory tract infections, excluding PNU	1.137 (29.5)	16 (17.4)
PNU	143 (1.4)	5 (5.4)
Skin infections, including SSI	894 (23.2)	28 (30.4)
Eye, ear, nose and mouth infection	183 (4.7)	8 (8.7)
Gastrointestinal infections, excluding CDI	75 (1.9)	4 (4.3)
CDI	37 (1.0)	2 (2.2)
Other infections	156 (4.0)	2 (2.2)
Prevalence of residents with at least one HAI [%]	3.7	3.9
Lack of microbiological results of HAIs testing* [%]	75.8	81.5

^*^Examination not done or result not available on the day of the PPS

*CDI* Clostridioides difficile infection, *HAI* healthcare associated infections, *PNU* pneumonia, *SSI* skin infections, including surgical site infections, *UTI* urinary tract infections

The lack of microbiological results of HAIs testing concerned the vast majority of all HAIs – 75.8% in total and 81.5% in Poland (Table 1).

The most frequently used group of antibacterials for systemic use was beta-lactams. The most frequently prescribed antimicrobial agent was “amoxicillin and enzyme inhibitor” (ATC code: J01CR02) 13.7% of all prescriptions in total and in Poland recorded in 26% of prescriptions. The next most common agents were nitrofurantoin (J01XE01; 9.5%) and trimethoprim (J01EA01; 9.0%). In Poland, instead of nitrofurantoin there was used only furazidin, which was applied in 16.4% of patients, while the share of trimethoprim/sulfamethoxazol was much smaller (4.1%) than in other countries (Table 2).

**Table 2:** Use of antibacterials for systemic use (ATC J01) used for prevention or treatment in Polish and all European group in Healthcare-Associated Infections in Long-Term Care Facilities Project (HALT-3), 2016–2017

Antibacterials for systemic use, anatomical therapeutic chemical classification	HALT PPS (n;%) [9]			
	Total		Poland	
	N (%)	AMC**	N (%)	AMC**
Tetracyclines, J01A	105 (2.1)	1.03	1 (1.4)	0.44
Amphenicols, J01B	2 (0.0)	0.02	0 (0.0)	0.00
Beta-lactams, penicillins, J01C	1515 (29.8)	14.81	20 (27.4)	8.77
Other beta-lactams, J01D	639 (12.6)	6.25	16 (21.9)	7.01
Sulfonamides and trimethoprim, J01E	724 (14.2)	7.08	3 (4.1)	1.32
Macrolides, lincosamides and streptogramins, J01F	369 (7.3)	3.61	3 (4.1)	1.32
Aminoglycosides, J01G	34 (0.7)	0.33	5 (6.8)	2.19
Quinolones, J01M	752 (14.8)	7.35	12 (16.4)	5.26
Combinations of antibacterials, J01R	9 (0.2)	0.09	0 (0.0)	0.00
Other antibacterials, J01X*	939 (18.5)	9.18	13 (17.8)	5.70
Total	5.088 (100.0)	49.74	73 (100.0)	32.00

*AMC* antimicrobial consumption, calculated by dividing the number of residents on antimicrobials on the day of survey by the total number of residents in LTCFs on the day of survey and multiplying by 100; *HALT PPS* Healthcare-Associated infections (HAIs) and antimicrobial use in Long-term care facilities Point Prevalence Survey; *n* number of prescriptions;

^*^Including: glycopeptides (J01XA), polymyxins (J01XB), steroid antibacterials (J01XC), imidazole derivatives (J01XD), nitrofuran derivatives (J01XE), others (J01XX)

^**^Antimicrobial consumption (AMC) calculated per 1000 residents, expressed as: (total number of residents on antimicrobials on the day of survey/total number of residents in LTCF on the day of survey) × 1000

^***^n/a non applicable

In order to analyze the causes of the low level of AMC in Poland, the relationship between AMC in treatment of patients in hospitals, outpatient clinics and at LTCFs, and the selected characteristics of the health care system utilization and demographics was examined. This included the access to professional medical care (expressed as the number of physicians and nurses per 1000 population) and the share of elderly people (aged 65+) in the general population (Additional file 2: Table S2).

The mean share of the elderly population in general population of all countries was 19.6% (standard deviation SD 2.1), while in Poland 16.7%. The mean number of nurses per 1000 inhabitants was in total 7.2% (SD 2.1), while in Poland 5.1%, and the mean number of physicians per 1000 inhabitants was in total 3.5% (SD 0.7), while in Poland 2.4%. The mean AMC in hospitals in total was 1.78 DDD per 1000 inhabitants per day, while in Poland 1.62 DDD per 1000 inhabitants per day. The mean AMC in community (ambulatory) care was respectively 18.3 and 23.8, expressed by DDD per 1000 inhabitants per day for a general group of countries and Poland, respectively [10].

The statistically significant relationships were found between AMC and selected demographic indicators. One of them was positive, the ratios of the elderly persons and physicians per population (rho = 0.575), and the second one was negative, for the availability to nurses’ care expressed as the number of nurses per 1000 inhabitants and the ratio of the elderly persons in the general population (rho = − 0.402). A statistically significant negative correlation was found for the share of the elderly population and AMC in LTCFs (rho = − 0.381), while the correlation concerning hospital AMC was statistically significant but positive (rho = 0.318). The correlation between the share of physicians per population and AMC in LTCF was positive and statistically significant (Table 3).

**Table 3:** Matrix of correlation of Spearman's rho coefficients for all countries participating in Healthcare-Associated infections and antimicrobial use in Long-term care facilities Point Prevalence Survey

Studied variables	Elderly population [%]	Nurses	Physicians	AMC		
		Per 1000 inhabitants		Hospital	Community	LTCF
Elderly population [%]	n/a	− 0.402*	0.575**	0.318*	− 0.01	− 0.381*
Nurses per 1000 inhabitants	− 0.402*	N/a	− 0.034	0.058	− 0.059	0.253
Physicians per 1000 inhibitants	0.575**	− 0.034	N/a	0.175	− 0.271	0.318*
Hospital AMC	0.318*	0.058	0.175	n/a	0.805**	0.12
Community AMC	− 0.01	− 0.059	− 0.271	0.805**	n/a	0.053
LTCF – AMC	− 0.381*	0.253	0.318*	0.12	0.053	N/a

*p < 0.05; **p < 0.01; AMC, Consumption of antibacterials (ATC group J01) for systemic use in the hospital/community sector (2017) and PPS (2016/2017)

*n/a* Not available

## Discussion

It was revealed in this study that the prevalence of HAIs in Polish LTCFs did not differ from that observed in similar settings in the EU, while the consumption of antibiotics was almost twice lower, which turned out to be a rather surprising finding, as the previous studies performed by the Authors pointed to the problem of high consumption of antibiotics within outpatient treatment in Poland [11].

Data on the use of antibiotics, the number of residents or patients of LTCF receiving oral, intravenous or intramuscular antibiotics (excluding topical preparations), and the incidence of infections were included in this study. A greater share of skin and subcutaneous tissue infections (for which the predominantly topical application of antimicrobials can be expected) was observed in the Polish population, in comparison with the European average, amounting to 30.4% vs. 23.2% respectively (Table 1). Furthermore, the share of eye, ear, nose and mouth infections in Polish group was almost twice higher than in all European countries. The relative frequency rates were 8.7% and 4.7% respectively (Table 1). Presumably, these are as well skin infection cases with predominant topical application of antimicrobials. Similarly, a higher share of gastrointestinal infections, for which symptomatic but not antimicrobial treatment is expected, was reported in Poland, in comparison with the European average—the corresponding rates were 4.3% vs. 1.9% (Table 1

In contrast, the prevalence rates for respiratory system infections (except pneumonia) were lower among the Polish residents/patients of LTFC than the European averages, i.e., 17.4% vs. 29.5% (Table 1). The reason for this could be the time of the study, i.e., June 2017 in Poland, while in other countries the study was conducted also in autumn, which is a season characterized in Europe with more frequently occurring respiratory infections, including those treated with antibiotics. The publicly available reports do not provide accurate data on the time frame and season over which the surveys were conducted in particular countries. These data (publicly available) also do not include the prevalence of antimicrobial usage according to site of infections or clinical form of infection, which is an important factor of its differentiation. In a cohort study of antimicrobial usage in US nursing homes, performed with application of the methodology similar to that applied in ECDC HALT-3, Thompson et al. [12] observed different schemes of antibiotic therapy depending on the site of infections, such as urinary tract, wound/skin, respiratory tract, bone or joint or gastrointestinal.

Similarly, as the prevalence rate for the respiratory system infections (except pneumonia) in LTCF, also the prevalence of urinary tract infections (UTI) was lower in Poland than in the general European study population. This finding could also help explain the relatively lower AMC in Poland. Moreover, a generally lower number of physicians (being the main prescribers of antimicrobial treatments) per population could partially explain the phenomenon of lower AMC in Poland, as compared with the situation in the general pool of European countries included in this study.

Additionally, the lower percentage of LTCF residents receiving antibiotics in the Polish group may be related to the fact that in the Polish LTCF participating in the study, as well as in the general population of the country, a smaller share of the oldest people was recorded. The average number of residents over 85 in the Polish group was 32.5%, and the median was 30.6 (based on unpublished data of ECDC from the preliminary report), while the corresponding indicators for the entire group (all participating countries) were 48.6% and 50.0%, respectively.

The calculated odds ratios (Table 2) for differences in prevalence of AMC of particular groups of antimicrobial agents between all countries participating in the study and Poland have not yielded the statistically significant results. Therefore, a study on a greater number of participants would have to be conducted, to determine whether there is a statistically and clinically significant difference in this aspect. If significant differences were discovered, they could prompt questions on what factors contribute to this potential disparity. As it was also mentioned in the summary of the HALT project, the type of survey chosen to collect data—a point prevalence survey, while being feasible to carry out, is prone to variation, since the situation at a specific moment may not represent the characteristics of healthcare institutions in a broader time range [7, 13].

A detailed analysis of the demographic structure and the available health care resources of the surveyed countries showed that low consumption of antibiotics is associated with a relatively young population, which is reflected in the low share of people aged 65 + in Poland.

The presented data indicate major problems regarding the antimicrobial consumption in also LTCF, even though the residents of Polish LTCFs are younger than observed in other EU countries. On the other hand, the contributor to increasing drug resistance is using antimicrobials without microbiology testing—in participating Polish LTCFs, according to the presented data, more than 80% of antimicrobial therapy was administered empirically, which happens to be more frequent than in the other countries.

## Strengths and limitations

The Authors must admit that the significant limitation of the analysis of relationship between selected AMC and demographic indicators and health care resources’ utilization rates was the availability of data from different countries, both ECDC and OECD. The data were fully available for Austria, Belgium, France, Hungary, Italy, Lithuania, Poland, Spain and the UK-Scotland. One of the limitations of the study is also the characteristics of the ECDC tool (HALT-3 protocol), which does not include data on the percentage of residents receiving hospital treatment in case of severe infections. However, this pertains to all participating countries, e.g., 17% of European population aged 65 + were hospitalised for at least one night in 2014, but this rate varies from country to country. In Poland it was very high, more than 20 days, similarly to Austria and Germany. However, in Greece it were less than 8% [14].

The current study is the first large multicenter study on the epidemiology of infections and the consumption of antibiotics in LTFCs in Poland. Such studies and analyzes have not been carried out so far in Poland.

## Conclusions

A significant problem observed in LTCFs in Poland and other European countries is the empirical use of antibiotics and the scarcity of microbiological testing. Surveillance of HAIs is a cornerstone for prevention and control of HAIs with antimicrobial stewardship program, but including microbiology surveillance with an early warning system. Microbiology testing as an element of antibiotic treatment and surveillance should be strengthened, both in LTCFs in Poland and throughout Europe. The antibiotic consumption in European LTCFs is closely correlated with the age of the population (65 +) and the availability of doctors. AMC in LTCFs was found to be lower in Poland than in the overall group of all studied European countries. In the studied Polish LTCFs, where the age of residents was low, the AMC was found to be lower than in the overall group of the studied European countries. It is advised to increase the number of doctors, corresponding to the expected growth of population aged 65 + in our country.

## Supplementary Information

Additional file 1: Table S1. The sources of data used for assessment of relationship between selected antimicrobial consumption, demographic and health care resources’ utilization rates.

Additional file 2: Table S2. Selected demographic, health resources and antimicrobial consumption (AMC) rates, EU countries, 2017

## Data Availability

The datasets generated or analyzed during this study are available and can be obtained, at request, from Anna Różańska (e-mail: a.rozanska@uj.edu.pl) on reasonable enquiry.

## Abbreviations

AMC: Antimicrobial consumption
ATC: Anatomical therapeutic chemical
EU: European Union
ECDC: European Centre for Disease Prevention and Control
HAI: Healthcare-associated infection
HALT PPS: HAIs in Long-Term care facilities Point Prevalence Survey
LTCF: Long-term care facilities
PNU: Pneumonia
