# Supplementary material for: Antibiotic consumption in long-term care facilities in Poland and other European countries in 2017
Source: Antimicrob Resist Infect Control. 2021 Oct 26;10:154. doi: 10.1186/s13756-021-01019-1 (PMC8549207; doi:10.1186/s13756-021-01019-1)
Supplement: Supplementary file 1 — The sources of data used for assessment of relationship between selected antimicrobial consumption, demographic and health care resources’ utilization rates. [file 13756_2021_1019_MOESM1_ESM.docx]

Additional file 1: Table S1. The sources of data used for assessment of relationship between selected antimicrobial consumption, demographic and health care resources’ utilization rates.

| Source of data: | |
| --- | --- |
| European Centre for Disease Prevention and Control (ECDC): | Organisation for Economic Co-operation and Development (OECD): |
| Characteristics of data and its application in this study | |
| For HALT PPS 2016-2017 [1]  The reports of the database are provided through the European Surveillance System (TESSy). | Elderly population 65+  The elderly population is defined as people aged 65 and over. The share of the dependent population is calculated as total elderly and youth population expressed as a ratio of the total population -OECD (2021)  Population (indicator) [3] |
| For hospitals and outpatient care consumption of antibiotics; data from the European Surveillance System—TESSy, provided by Austria, Belgium, Croatia, Estonia, Finland, France, Greece, Hungary, Ireland, Italy, Latvia, Lithuania, Malta, the Netherlands, Poland, Slovenia, Spain and the UK–Scotland, and released by ECDC [2] | Health resources:  Physicians are defined as "practising" physicians providing direct care to patients. Physicians are usually generalists who assume responsibility for the provision of continuing care to individuals and families, or specialists such as paediatricians, obstetricians/gynaecologists, psychiatrists, medical specialists and surgical specialists. This indicator is measured per 1,000 inhabitants - OECD (2021)  Doctors (indicator) [4] |
|  | Health care resources:  Nurses are defined as all the "practising" nurses providing direct health services to patients, including self-employed nurses. Midwives and nursing aides (who are not recognised as nurses) are normally excluded although some countries include midwives as they are considered specialist nurses. This indicator is measured per 1,000 inhabitants - OECD (2021)  Nurses (indicator) [5] |

**Bibliography:**

1. Database available at (25.03.21): <https://www.ecdc.europa.eu/en/all-topics-z/healthcare-associated-infections-long-term-care-facilities/surveillance-and-disease-11>
2. European Centre for Disease Prevention and Control. Antimicrobial consumption in the EU/EEA – Annual Epidemiological Report 2019. Stockholm: ECDC; 2020 Available at (07.06.21): https://www.ecdc.europa.eu/sites/default/files/documents/Antimicrobial-consumption-in-the-EU-Annual-Epidemiological-Report-2019.pdf
3. OECD (2021), Population (indicator). (Accessed on 16 February 2021) <https://data.oecd.org/pop/population.htm>
4. OECD (2021), Doctors (indicator). (Accessed on 16 February 2021) <https://data.oecd.org/healthres/doctors.htm#indicator-chart>
5. OECD (2021), Nurses (indicator). (Accessed on 16 February 2021) <https://data.oecd.org/healthres/nurses.htm#indicator-chart>
