# Supplementary material for: Antibiotic consumption in long-term care facilities in Poland and other European countries in 2017
Source: Antimicrob Resist Infect Control. 2021 Oct 26;10:154. doi: 10.1186/s13756-021-01019-1 (PMC8549207; doi:10.1186/s13756-021-01019-1)
Supplement: Supplementary file 2 — Selected demographic, health resources and antimicrobial consumption (AMC) rates, EU countries, 2017 [file 13756_2021_1019_MOESM2_ESM.docx]

Additional file 2: Table S2

|  | |  | |  |  | |  | |  |  |  |
| --- | --- | --- | --- | --- | --- | --- | --- | --- | --- | --- | --- |
| EU Country | Consumption of antibacterials (ATC group J01) for systemic use in the hospital/community sector (2017) | | | | | PPS (2016/2017) | | Health resources | | | Elderly population 65+ |
|  | Hospital | | Community | | | LTCF | | Nurses | | Physicians |  |
|  | DDD* per 1000 inhabitants and per day | | | | | prevalence | | total, per 1000 inhabitants | | | per 100 inhabitants |
| Austria | - | | 11,90 | | | 3,20 | | 6.9 | | 5.2 | 18.6 |
| Belgium | 1,64 | | 21,10 | | | 5,90 | | 11.2 | | 3.1 | 18.6 |
| Croatia | 1,74 | | 16,80 | | | 2,00 | | - | | - | 19.8 |
| Estonia | 1,65 | | 9,90 | | | - | | 6.3 | | 3.5 | 19.5 |
| Finland | 2,11 | | 13,60 | | | 6,70 | | - | | - | 21.7 |
| France | 2,13 | | 29,20 | | | 2,70 | | 10.8 | | 3.4 | 19.4 |
| Greece | 2,29 | | 37,40 | | | 6,00 | | - | | - | 21.7 |
| Hungary | 1,24 | | 15,60 | | | 0,90 | | 6.6 | | 3.3 | 18.8 |
| Ireland | 1,60 | | 19,30 | | | 9,70 | | - | | 3.3 | 13.6 |
| Italy | 1,89 | | 19,00 | | | 4,30 | | 6.7 | | 4.0 | 22.4 |
| Latvia | 1,89 | | 12,10 | | | - | | 4.3 | | 3.2 | 20.0 |
| Lithuania | 2,12 | | 13,60 | | | 0,70 | | 7.8 | | 4.6 | 19.5 |
| Malta | 3,11 | | 23,40 | | | 2,70 | | - | | - | 18.8 |
| Netherlands | 0,94 | | 10,10 | | | 4,40 | | - | | - | 18.7 |
| Poland | 1,79 | | 27,00 | | | 3,20 | | 5.1 | | 2.4 | 16.7 |
| Slovenia | 1,44 | | 10,70 | | | - | | 10.1 | | 3.1 | 19.1 |
| Spain | 1,83 | | 25,10 | | | 10,50 | | 5.9 | | 3.9 | 19.1 |
| UK-Scotland | 2,62 | | 19,10 | | | 6,40 | | 7.8 | | 2.8 | 18.2 |
| Mean (SD) | 1,8 (0,50) | | 18,7 (8,02) | | | 4,7 (3,01) | | 7.2 (2.1) | | 3.5 (0.7) | 19.6 (1.4) |
| MD (Q1;Q3) | 1,8 (1,60;2,12) | | 17,3 (12,00;24,25) | | | 4,3 (2,70;6,00) | | 6.7 (6.1;6.8) | | 3.3 (3.1;3.9) | 19.4 (18.8;19.9) |

Table S2. Selected demographic, health resources and antimicrobial consumption (AMC) rates, EU countries, 2017

References:

1. OECD (2021), Nurses (indicator). doi: 10.1787/283e64de-en (Accessed on 16 February 2021) <https://data.oecd.org/healthres/nurses.htm#indicator-chart>
2. OECD (2021), Doctors (indicator). doi: 10.1787/4355e1ec-en (Accessed on 16 February 2021) <https://data.oecd.org/healthres/doctors.htm#indicator-chart>
3. OECD (2021), Population (indicator). doi: 10.1787/d434f82b-en (Accessed on 16 February 2021) <https://data.oecd.org/pop/population.htm>
4. OECD (2021), Elderly population (indicator). doi: 10.1787/8d805ea1-en (Accessed on 16 February 2021) <https://data.oecd.org/pop/elderly-population.htm#indicator-chart>
